# Gut Microbiota as a Therapeutic Target to Ameliorate the Biochemical, Neuroanatomical, and Behavioral Effects of Traumatic Brain Injuries

**DOI:** 10.3389/fneur.2019.00875

**Published:** 2019-08-16

**Authors:** Matthew W. Rice, Jignesh D. Pandya, Deborah A. Shear

**Affiliations:** Brain Trauma Neuroprotection Branch, Center for Military Psychiatry and Neuroscience, Walter Reed Army Institute of Research, Silver Spring, MD, United States

**Keywords:** traumatic brain injury, therapy, gut microbiome, microbiota-gut-brain axis, gut dysbiosis

## Abstract

Current efficacious treatments for traumatic brain injury (TBI) are lacking. Establishment of a protective gut microbiota population offers a compelling therapeutic avenue, as brain injury induces disruptions in the composition of the gut microbiota, i.e., gut dysbiosis, which has been shown to contribute to TBI-related neuropathology and impaired behavioral outcomes. The gut microbiome is involved in the modulation of a multitude of cellular and molecular processes fundamental to the progression of TBI-induced pathologies including neuroinflammation, blood brain barrier permeability, immune system response, microglial activation, and mitochondrial dysfunction, as well as intestinal motility and permeability. Additionally, gut dysbiosis further aggravates behavioral impairments in animal models of TBI and spinal cord injury, as well as negatively affects health outcomes in murine stroke models. Recent studies indicate that microbiota transplants and probiotics ameliorate neuroanatomical damage and functional impairments in animal models of stroke and spinal cord injury. In addition, probiotics have been shown to reduce the rate of infection and time spent in intensive care of hospitalized patients suffering from brain trauma. Perturbations in the composition of the gut microbiota and its metabolite profile may also serve as potential diagnostic and theragnostic biomarkers for injury severity and progression. This review aims to address the etiological role of the gut microbiome in the biochemical, neuroanatomical, and behavioral/cognitive consequences of TBI, as well as explore the potential of gut microbiome manipulation in the form of probiotics as an effective therapeutic to ameliorate TBI-induced pathology and symptoms.

## Brief Overview of Traumatic Brain Injury

Traumatic brain injury (TBI) is a major cause of death and disability in the United States and represents one of the most prevalent injury types sustained by the worldwide population (1). Reports spanning the last two decades underscore the human and financial burden of TBI in the United States, with an annual incidence of ~1.4 million cases (2), prevalence of ~3.17 million with a long-term TBI-induced disability (3), and an annual economic burden of billions of dollars (4). Importantly, these disabilities are a result of not only the mechanical damage sustained due to the initial injury (primary), but also the subsequent cellular and molecular damage that exacerbates in the following hours, days, weeks, and years post-injury (secondary) (5, 6). The etiology of secondary injury is multifaceted and may constitute altered cerebral blood flow, excitotoxicity, inflammation, microglial activation, metabolic anomalies, mitochondrial dysfunction, and oxidative stress resulting in transient or lifelong behavioral and cognitive deficits (5–9). TBI severity is categorized based on the Glasgow Coma Scale (GCS), in which patients are scored on the basis of clinical symptoms, and the resulting overall score classifies their injury as mild (score: 13–15), moderate (score: 9–12), or severe (score: <9) (10, 11). Overall, TBI complexity occurs on a spectrum ranging from mild to severe, diffuse to focal, and single to repeated exposures in brain vs. multi-organs, which leads to injury-specific heterogeneous pathobiological responses that cannot be regarded as a single condition (12).

Despite decades of rigorous preclinical research in which much insight into the heterogeneous nature of brain injury has been gained, efficacious therapeutics for TBI-induced neuropathologies and behavioral/cognitive impairments are lacking (13–15). Given the prevalence of TBI-related disabilities, it is imperative to consider novel treatment strategies. Restoration of the gut microbiome by gut eubiotic therapeutics is one such compelling avenue, which is capable of modulating the bi-directional relationship between TBI-induced disruptions of the gut microbiome and the influence of this gut dysbiosis on the pathophysiology of TBI-induced secondary injury progression (16, 17).

## Microbiota-Gut-Brain Axis (MGBA)

Gut microbiota refer to the bacteria, archaea, viruses, and eukaryotic microbes that reside primarily within the colon, but also within the stomach and small intestine (18). This commensal bacterial community accounts for 0.2–1 kg of an adult's bodyweight (18, 19), outnumbering mammalian cells by as much as 10:1, though more recent estimates indicate a ratio of ~1:1 (18), and contains ~100 fold more unique genes than the human genome (20). Bacteroidetes and Firmicutes phyla compose the majority of the gut microbiota, with Proteobacteria, Actinobacteria, Fusobacteria, and Verrucomicrobia being present in fewer numbers. However, gut microbiota composition differs among individuals as diet, age, gender, environment, and genetics all influence bacterial strains/populations (21–23). The activity and composition of this microbial population is involved in a surprising number of biological processes, including homeostasis of the central nervous system (CNS) (24–26). This relationship is referred to as the microbiota-gut-brain axis (MGBA) (27), with communication between the gut microbiota and the CNS occurring through a neuro-endocrino-immunological network (28).

Perhaps the most direct route of communication within the MGBA is among the gut microbiota, enteric nervous system (ENS), and vagus nerve. Neuroactive compounds produced by gut bacteria influence the activity of sensory neurons of the ENS, which in turn modulates the afferent activity of the vagus nerve (29). These compounds consist of bacterial metabolites, neurotransmitters, neurotrophic factors, cytokines, and endotoxins (30–32). Nervous system signaling originating from the gastrointestinal tract is then integrated by the nucleus of the solitary tract (33) and relayed to other brain nuclei (34). Gut microbiota also play a fundamental role in the development and functioning of the host immune system (35). Homeostasis of host immune system function is predicated upon proper gastrointestinal neuromuscular control, maintenance of intestinal wall integrity, and intact ENS/vagus nerve signaling (36, 37), aspects of gastrointestinal health that are, in part, regulated by the gut microbiome. Perturbations in the composition of the gut microbiota are known to lead to a weakening of the intestinal-host barrier (38), allowing gastrointestinal content to be released into the blood stream and other parts of the body, a condition referred to as “leaky gut” (39), which can lead to neuroinflammation. For example, peripheral administration of the bacterial endotoxin lipopolysaccharide induces cytokine expression within the hypothalamus-pituitary-axis, resulting in regional neurotoxicity and systemic inflammation (40, 41). Notably, the cross-talk among the gut bacteria, ENS, and vagus nerve cohesively regulates the host immune and inflammatory responses to modulate CNS function (42, 43). Finally, cognitive and behavioral changes (e.g., stress) have repeatedly been shown to alter the composition of the gut microbiota, demonstrating both feed-forward and feedback mechanisms within the MGBA (44).

Gut microbiome composition has been linked to a variety of illness and disease states (45, 46), with research dating back over seven decades establishing a relationship between the metabolic products of gut bacteria and hepatic encephalopathy (47, 48). More recent research has linked the gut microbiota to inflammatory diseases (49) and several CNS-related disorders, including autism (50, 51), depression (28, 52), and anxiety (53, 54), as well as Alzheimer's disease (55) and Parkinson's disorder (55, 56). However, it is difficult to prove causation and directionality when discussing gut microbiome changes observed in human neuropsychiatric and neurodegenerative conditions (57). For these reasons, rodents are commonly used when investigating the MGBA as they (1) possess similar, but not identical, core intestinal bacterial populations to humans (58, 59) and (2) can be maintained “germ free” (devoid of gut microbiota) or gnotobiotic (gut microbiota of known composition).

Eubiotic therapeutics that alter the gut microbiome through diet, microbiota transplants, antibiotics, and pre-/probiotics influence both systemic and CNS-related processes. Microbiota transplants have been shown to influence obesity levels in rodents (60) and humans (61), as well as effectively treat recurrent *Clostridium difficile* infection (62). Meanwhile, probiotics have shown promise in the treatment of patients with ulcerative colitis (63) and antibiotics are now commonly used to eliminate the bacterial populations involved in hepatic encephalopathy (64). Probiotics have also been shown to reduce anxiety- and depressive-like symptoms in animals, with limited evidence indicating similar results in humans (53). Furthermore, gut microbiome alterations have been shown to ameliorate autism-like behaviors in mice (65), with probiotics having been suggested as a therapeutic strategy for individuals with post-traumatic stress disorder (66). Among other research findings [as reviewed by (67)], this has led some researchers to suggest “psychobiotics” as a new therapeutic approach for neurological and neuropsychiatric illnesses (68, 69).

## Role of the MGBA in CNS Injuries

**Figure 1:**
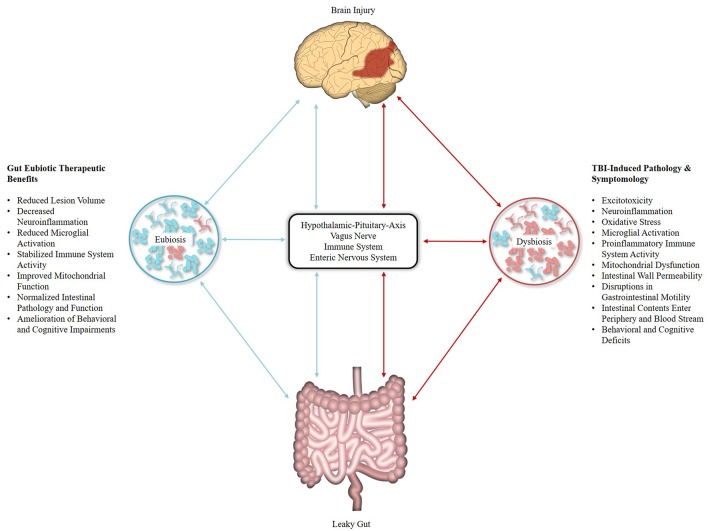
Effects of traumatic brain injury (TBI) and eubiotic therapies within the microbiota-gut-brain axis (MGBA). Brain injury induces disruptions within the MGBA through multiple pathways [Represented in Red]. Resulting perturbations complete a bi-directional positive feedback mechanism that contributes to the secondary injury characteristics of TBI. Resolution of gut dysbiosis by eubiotic therapeutics may act to break this cycle [Represented in Blue], thus reducing the impact of secondary injury pathology and improving TBI biochemical, pathological, and behavioral outcomes.

Pertinent for TBI research is the bi-directional relationship that exists between brain injury and the gut microbiome (Figure 1). Research in brain and spinal cord injury (SCI) animal models has demonstrated that CNS injury disrupts the motility and permeability of the intestinal wall (70, 71) and perturbs the composition of the gut microbiome (17, 72), leading to a host-maladaptive state referred to as gut dysbiosis (73). Conversely, gut dysbiosis influences the pathophysiology of traumatic CNS injury (74, 75). For example, following SCI, significant changes in the composition of the gut microbiota were observed, namely a decrease in Bacteroidetes and increase in Firmicutes, with post-injury changes in the gut microbiome persisting out to 1 month and predicting the degree of locomotor impairment (76). A similar relationship was observed in a controlled cortical impact (CCI) rodent model of moderate TBI, with bacterial changes occurring as early as 2 h following injury, persisting out to 7 days post-injury, and correlating with lesion volume. However, the opposite alteration in gut microbiota was observed with a decrease in Firmicutes and increase in bacterial families within the Bacteroidetes and Proteobacteria phyla (77). Furthermore, a recent study by Treangen et al. (78) reported gut dysbiosis with significant decreases in *Lactobacillus gasseri, Ruminococcus flavefaciens*, and *Eubacterium ventriosum* and significant increases in *Eubacterium sulci* and *Marvinbryantia formatexigens* at 24 h post-CCI in mice. *L. gasseri* displayed the most drastic change with a 4-fold log decrease in abundance as compared to baseline, though it should be noted that a less pronounced decease was also observed following sham procedures. As *L. gasseri* is a member of the phylum Firmicutes, this work complements the findings of Nicholson et al., and provides for a potential eubiotic target as *L. gasseri* inhabits the human gut microbiome (79). Investigations into TBI-induced gut dysbiosis in humans is limited, though a recent study in severely injured patients with polytrauma reported a decrease in Bacteroidales, Fusobacteriales, and Verrucomicrobiales, as well as an increase in Clostridiales and Enterococcus within 72 h of injury (80).

Gut dysbiosis also affects the integrity and permeability of the blood brain barrier (BBB) (81). Coupled with TBI-induced physical disruptions to the BBB (82), intestinal contents and the associated upregulation of the pro-inflammatory immune response more easily permeate the CNS, resulting in increased microglial activity, neuroinflammation, and neuropathology (83, 84). Microglial maturation and function within the CNS have been shown to be influenced by the gut microbiome in BBB-intact animals (85, 86), a relationship expected to be enhanced by increased BBB permeability. Therefore, it is likely that TBI-induced gut dysbiosis is a contributing factor in increased microglial activation following CNS injury (86). Post-injury mitochondrial dysfunction in terms of energy production (i.e., ATP synthesis) observed in TBI (87, 88) may also be impacted by gut dysbiosis, as studies have revealed a link between gut bacterial metabolites and mitochondrial function (26, 89).

Importantly, experimenter-induced alterations in the composition of the gut microbiota community regulate immune system activity, neuropathology, and behavior following CNS injury. In a gnotobiotic mouse model of ischemic stroke, an expansion of Proteobacteria accompanied by a contraction in Firmicutes and Bacteroidetes altered immune system homeostasis by increasing peripheral neuroprotective anti-inflammatory T_reg_ cells and decreasing pro-inflammatory γδ T cells, resulting in a reduction in ischemic brain injury (90). However, the large-scale depletion of cultivatable gut microbiota by a broad-spectrum antibiotic in a mouse model of focal cerebral ischemia prior to injury resulted in decreased rates of survival and an increase in the development of severe acute colitis (74). Furthermore, if gut dysbiosis was experimentally induced by a broad-spectrum antibiotic prior to SCI, both neurological impairment and spinal cord pathology were exacerbated, likely due to changes in immune system activity (76). These studies demonstrate the complex relationships within the MGBA, revealing that the bacterial populations present at the time of injury influence the degree of neuropathology and functional impairment following TBI. Such knowledge establishes the basis for both the monitoring and manipulation of the gut microbiota as a means to diagnose and ameliorate the pathophysiology and symptomology of brain injuries.

## Gut Microbiota as a Potential Diagnostic and Therapeutic Target for TBI

Monitoring the extent of gut dysbiosis may provide a diagnostic tool for the identification of TBI severity, providing information for treatment guidance. Fecal metabolomes have already been used as biomarkers for several ailments including Crohn's disease and colorectal cancer (91, 92), and a recent study by Houlden et al. (72) demonstrated a positive correlation between the degree of gut dysbiosis and the severity of a closed-head-impact rodent model. Importantly, the profile of gut microbiota changes observed following TBI differed from those following ischemic brain injury by 72 h post-injury, indicating that different forms of brain injury uniquely impact the gut microbiome (72).

Beyond monitoring, manipulation of the gut microbiome via eubiotic therapies (e.g., microbiota transplants and pre/probiotics) presents an exciting treatment target for TBI (Figure 1). Several of the ailments associated with TBI-induced pathology that affect the microbiota are improved by the intake of probiotics, such as intestinal motility and permeability, health of the intestinal cellular lining, intestinal inflammation, and systemic immune response (93–95). Furthermore, perturbations in bacterial composition initially appear 24–72 h following trauma (72, 80); a time period corresponding to the pathophysiology of TBI-induced secondary injury, representing an ideal treatment window. As substantial alterations in the gut microbiome can occur 24–48 h following dramatic changes in diet (96, 97), eubiotic therapies could fundamentally shift the gut microbiome to a beneficial state in time to mitigate aspects of TBI-associated secondary injury. Preclinical studies support this concept as microbiota transplants have been shown to reduce brain lesion size and improve health outcomes in mouse models of ischemic stroke (98) and restore microglial function (85). Probiotic derived bacterial metabolites may also serve to modulate mitochondrial homeostasis (99) as gut microbiota generate short-chain fatty acid products such as butyrate, propionate, and acetate (100). Together with dietary ketones, these gut microbiome products serve as alternative energy sources for the injured brain and may improve bioenergetics function following TBI and SCI (101, 102). Additionally, gut microbiota-generated butyrate serves as a histone deacetylation (HDAC) inhibitor, offering additional benefits as HDACs play an important role in neuroprotection following CNS injuries (103) and enhance cognitive function in neuropsychiatric disorders (104). Furthermore, the butyric acid-producing probiotic *Clostridium butyricum* improved neurological deficits, reduced brain edema, attenuated neurodegeneration, and ameliorated BBB impairment (105), as well as improved spatial memory in mouse models of weight-drop impact head injury and cerebral ischemia, respectively (83). Probiotic supplements rich in lactobacilli and bifidobacteria have also been shown to improve spatial memory in a cognitively impaired mouse model (106) and one explanation for these observed improvements is evidenced by VSL#3 (a commercial, medical-grade probiotic rich in lactic acid bacteria) rescuing hippocampal neurogenesis via Ly6C^hi^ monocytes in mice with antibiotic-induced gut dysbiosis (107). Treatment with VSL#3 also decreases circulating levels of TNFα, lessens cerebral monocyte infiltration, and reduces microglial activation (108). In mice that received SCI, VSL#3 provided the day of injury and extending for 35 days post-injury reduced neuropathology, improved locomotor recovery, and triggered a protective immune response through an increase in the number of T_reg_ cells (109).

Importantly, human preclinical trials in brain injury patients with GCSs of 5–12 (i.e., moderate to severe TBI) indicate that manipulation of the gut microbiome through lactobacilli-rich probiotic supplementation within the first 48 h of admission with continued treatment for between 5 and 21 days can reduce nosocomial infection rate (110), decrease gastrointestinal dysfunction (111), lessen the incidence of ventilator-associated pneumonia (111), and shorten the time spent in intensive care (112). These observed benefits are commonly attributed to probiotic-induced reductions in systemic and central inflammation (113, 114). No studies exist examining the behavioral/cognitive outcomes of probiotic supplementation on TBI patients; however, probiotics have been shown to improve behavior and cognition in individuals with Alzheimer's disease (115) and depression (116), as well as healthy individuals (117). Probiotic supplementation for patients with penetrating TBI may be additionally useful as the long-term use of antibiotics is recommended for the reduction of infection, morbidity, and mortality rates (118, 119). As discussed, antibiotic-induced disruptions of the gut microbiome can lead to worsened TBI-related outcomes, potentially guiding medical practices toward adjunctive probiotic treatments to mitigate or minimize complex downstream pathobiological responses following TBI.

## Conclusion

Provided the bi-directional relationship between the gut microbiome and TBI-associated pathology, resolution of gut dysbiosis represents a compelling therapeutic target. Probiotics consisting of lactobacilli, bifidobacteria, and other butyrate-producing gut bacteria appear most beneficial, providing a eubiotic therapy that enhances MGBA function through their anti-inflammatory and positive mitochondrial energetic properties. However, recent work revealed that antibiotic-induced microbiome perturbations and probiotic colonization display strong inter-species and inter-individual differences that may not have been apparent in previous investigations (120, 121). Additionally, differing courses/compositions of eubiotic treatments may need to be considered based on the type and severity of CNS injury, as these parameters produce dissimilar gut dysbiosis profiles (72). Therefore, resolution of gut dysbiosis as a therapeutic option requires investigations that yield information on the specific changes that occur to the gut microbiota following different types and severities of TBI, as well as optimal doses, treatment window, duration of treatment, and efficacy of experimentally-induced gut microbiome alterations across age and gender. Data that are sorely lacking (17, 93). Ultimately, this information could be used to develop a powerful diagnostic tool or eubiotic therapy to alleviate trauma brought on by brain injury.

## Author Contributions

MR wrote the manuscript. JP contributed to manuscript revision. DS read and approved the submitted version.

### Conflict of Interest Statement

The authors declare that the research was conducted in the absence of any commercial or financial relationships that could be construed as a potential conflict of interest.
